# Supplementary material for: Ports’ criticality in international trade and global supply-chains
Source: Nat Commun. 2022 Jul 27;13:4351. doi: 10.1038/s41467-022-32070-0 (PMC9327979; doi:10.1038/s41467-022-32070-0)
Supplement: Supplementary file 1 — Supplementary Information [file 41467_2022_32070_MOESM1_ESM.pdf]

Supplementary Information

Ports' criticality in international trade and global supply-chains

J.Verschuur[1,*], E.E. Koks[1,2] and J.W. Hall[1]

[1] Environmental Change Institute, University of Oxford, Oxford, United Kingdom
[2] Institute for Environmental Studies, Vrije Universiteit Amsterdam, Amsterdam, Netherlands
[*]corresponding author: jasper.verschuur@keble.ox.ac.uk
Environmental Change Institute, 3 South Parks Road, OX1 3QY, Oxford, United Kingdom

Contains

Supplementary Notes 1-3
Supplementary Figures 1-11
Supplementary Tables 1-8
Supplementary References

Supplementary Note 1: Modal split model

We develop a global modal split (or modal choice) model to predict the share of maritime trade in every bilateral trade flow. A model split model intends to predict the allocation of freight transport flows for a given Origin-Destination (O-D) pair provided with alternative and competing transport modes[1]. We fit the model based on reported modal share data of international trade from UN Comtrade[2], which includes reported modal split for around 50 countries that report this data. Afterwards, we predict the model split in every bilateral trade flow reported in the harmonized BACI trade database[3], which the most comprehensive database of historical trade flows. We use 2015 as our base year as it is the latest available year (at the time of writing) available in the EORA MRIO database.

We consider maritime, air and land (road and rail) transport as alternative transport modes, given nearly all trade in our sample is by means of these three modes. We adopt a multinomial logit formulation, as is common practise into transport modelling[1,4,5], which is based on the concept of utility maximisation given a set of alternative modes, which have mode-specific variables (e.g. distance, time), as well as characteristics of importing and exporting country (e.g. income level, island, neighbouring countries), and the commodity (e.g. quantity, value to weight ratio, perishable or not). The model is set up to predict the share of maritime trade given the availability of maritime, air and (if possible) land transport.

In general, one can write the utility of a given mode (m) for commodity (c) as:

$$U_{m,c} = \beta_1 C_m + \beta_2 t_m + \beta_i U_m + \beta_i V_c + M_m$$

With *C* being the transport costs (USD per tonnes), *t* being the transit time (hours), *U* being a set of explanatory variables of the country pairs but irrespective of the commodity (GDP per capita of origin and destination, neighbouring country dummy, island dummy), *V* being a set of explanatory variables related to the commodity being shipped (quantity, value per tonnes, perishable dummy), and *M* being a mode-specific constant.

The cost function we adopt includes both the distance costs and the time costs, which was found to be the best performing formulation in previous work[6]. The total costs per mode (*C*) can be written as:

$$C_m = D_{c,m} d_m + T_{c,m} t_m + H_{c,m}$$

With D_c being the distance costs (USD per tonnes-km), *d* the distance (km), T_c the time costs (USD per tonnes-hour), H_c the handling costs (USD per tonnes), and *t* the transit time (hours). The time component is added in the cost function as well as a separate variable (β_2), which reflects that changes in the transit time not only results in capital costs, but also other value of time costs related to depreciation, inflation and insurance[6]. For some commodities, this can be an important additional factor to decide upon a certain transport mode (e.g. for time sensitive goods).

Per origin and destination flow, and per commodity, the share per mode (S) can be estimated using the multinomial mode choice probability[7]:

$$S_{m,c} = \frac{e^{U_{m,c}}}{\sum_{j \in m} e^{U_{j,c}}}$$

With m the number of mode alternatives, which is three for country pairs where land transport is available and two for country pairs where this is not.

Observed modal share data is collected from UN Comtrade[2] for the period 2016-2018, which, for the countries that report this, describes per bilateral trade flow on a commodity level (HS6). the share of different modes of transport being used. We filter out trade flows between non-landlocked countries in order to avoid misclassification of the mode of transport (e.g. Switzerland reports trade from Argentina as being road, because it uses road transport to enter the country) and remove trade flows that are specified as road, but where no road connection is present (e.g. trade from Brazil through the Port of Rotterdam to Germany is classified as road or rail). This results in 6.8 million bilateral trade flows between ~12,000 unique country pairs.

To fit the modal split model, mode-, country- and commodity-specific data was collected. For the country-specific data, we use GDP per capita from the World Development Indicators database[8], neighbouring country dummies based on the GeoDist database[9], and island country dummies, which are found by extracting countries without any neighbouring countries in the Geodist dataset. Commodity-specific data (on HS6 level) is collected from the BACI trade database[3], from which we extract the total quantity of trade flows and the value to weight conversion. Perishable product dummies are taken from the database provided by Hummels and Schaur[10].

Mode-specific data per country pair includes the aggregated cost and time required to ship goods between both countries. To derive this, we create a global transport network for air, maritime and land-based transport modes. Per transport mode, we add information on the distance, speed, handling cost, and additional dwell times (e.g. loading and unloading time and dwell time at port, airport, land border) to the network. As origin and destination locations, we use the centroid of countries derived in earlier work[11], in which nightlight data was used as a suitable weight to create country centroids.

For land-based transport, a global road transport network is extracted from the gROADSv1 global road database[12], while for the rail transport network OpenStreetMap (OSM) railway data is used. For both networks, we create a routable unidirectional network connecting the road/rail segments. At the borders, a border crossing time is added, which resembles border compliance processes (e.g. customs, etc). Border compliance time per country is taken from the World Bank's Doing Business database[13], which is added to every border crossing between two countries by taking the average of the two countries' border compliance time. For every road

and rail segment, we add a speed proxy per country, for which we distinguish between two groups of countries in line with Martínez et al.[14] and which are summarised in Supplementary Table 3. Moreover, we add distance and time costs to every segments, as well as handling costs and loading/unloading times at nodes where goods are transhipped from one mode to the other (e.g. centroid to road, road to rail, etc). The distance costs are taken from a database of mode specific distance costs for country globally provided by the ITF-OECD[14]. Time costs, handling costs and (un)loading times are taken from the literature and included in Supplementary Table 4-7. We run lowest cost paths between all centroids using Dijkstra shortest path algorithm[15], and derive the time associated with the lowest cost route. If both road and rail connections are feasible, we use the lowest cost of both modes as the representative land-based shipping costs.

For air transport, we create a global network of flight connections between airports. To do this, we combine data on passenger flight connections from OpenFlight database (https://openflights.org/data.html) and a global air cargo network (based on data of four major air cargo freight integrators)[16]. We connect country centroids to the airports via the road transport network, and add a dwell time at the airports (time that goods spend at an airport) and a transhipment dwell time (in case there is no direct flight connection), which are set based on recommended values in previous research[14,17]. All parameters adopted are summarised in Supplementary Table 4,5. Moreover, we add distance, time and handling costs to the network (see Supplementary Table 5-7), after which estimate the cost and transit time per country pair using the shortest path algorithm.

To estimate the maritime transport costs and times, we create a maritime transport network based on Oak Ridge National Laboratory maritime transport network[1], which we connect to a set of 1450 ports globally (see Supplementary Note 3) and consequently to the road transport network. In addition, we create a database of ports that are connected to one another based on observed port visits from ship movement data (Automatic Identification System, AIS) for the period 2019 – 2020 (see Supplementary Note 3). Hence, flows can only happen between ports that are connected to each other in the transport network. Several dwell times are added to this network. First, if a port in a neighbouring country is used, the border cross time is added in line with the land transport network. Second, at every port, we add a turnaround time of vessels based on the median turnaround time derived from AIS data over the period 2019 – 2020. Third, we add a cargo dwell time and transhipment dwell time to the ports, which are based on previous work[14] and summarised in Supplementary Table 5. After adding distance, time and handling costs to the network, we derive the maritime shipping costs and time per country pair (see Supplementary Tables 5-7.

We fit a separate modal split model for the 11 sectors adopted in this work to capture sector-specific model parameters. We perform a validation of the data on a sector level, with scatterplots included in Supplementary Fig. 10. For the maritime modal share, the regression models have a R-squared values ranging between 0.36 – 0.52, while the final maritime trade validation yields R-squared values ranging between 0.41 – 0.99.

---

[1] https://tedb.ornl.gov

To create a harmonized maritime trade dataset, we fit the model with the entire 2015 BACI harmonized trade dataset[3], which comprises commodity-specific (HS6) trade data on 8 million bilateral trade flows between ~24,000 unique country pairs. We use the 2015 trade data for this prediction given that we couple this data to the EORA MRIO table, which has 2015 as its most recent year. As additional validation, compare the predicted maritime shares for the United States, Australia, New Zealand and Europe to reported numbers, which show an overall good result (Supplementary Note 2).

Supplementary Note 2: Validation modal split model

Apart from the internal validation process, we perform an external validation of the results for countries for which we could find modal share data. For New Zealand, we find that maritime transport accounted for 81.4% of imports and 90.9% of exports in 2015, whereas the official data (average of 2005-2015)[2] showed that these shares were 77% and 88%, respectively. In Australia, 84.5%[3] of all trade by value is maritime, which is in line with our prediction of 82.2% of trade in terms of value. For the United States, we find maritime import and export shares of 55.3% and 51.5%, respectively, which are in line with the number provided by the United States Department of Transportation who estimated these shares to be 53% and 38% in 2011[4]. The overestimation of exports can be caused by the different reference years used. Moreover, we underestimation the amount of land transport between the U.S.A. and Canada and Mexico, which is likely due to relatively large distance between the centroids of these countries. For the European Union (EU) countries, the share of maritime transport in extra-EU trade was 53.0% for imports and 48.1% for exports in 2015[5]. Our model prediction estimates these shares to be 45.4% for imports and 45.6% for exports in 2015. Our models overestimates the amount of goods entering Europe by means of land transport, particularly from Asia, where additional factors cause shippers to favour maritime transport over land-based transport. Moreover, we can estimate the accuracy per country for the EU28 countries. The prediction is well, with correlation coefficients of 0.77 (exports) and 0.65 (imports).

---

[2] http://archive.stats.govt.nz/browse_for_stats/industry_sectors/imports_and_exports/overseas-merchandise-trade/Methods-transporting-imported-and-exported-goods.aspx#gsc.tab=0
[3] https://shippingaustralia.com.au/wp-content/uploads/2020/11/SAL20048-FACT-SHEET-ON-AUSTRALIAN-TRADE-by-SAL-1.pdf
[4] https://www.bts.gov/archive/publications/by_the_numbers/maritime_trade_and_transportation/index
[5] https://ec.europa.eu/eurostat/statistics explained/index.php/International_trade_in_goods_by_mode_of_transport

Supplementary Note 3: OxMarTrans model

This Supplementary Note describes the development of the new global maritime transport model, the OxMarTrans model, which simulates the allocation of maritime trade flows between subnational units globally on the maritime transport network. Previous research have made significant progress in developing global maritime transport models. Most notably are those developed by Tavasszy et al.[18], who constructed a network flow model to predict container flows between 437 container ports globally (with no explicitly hinterland representation), and Martinez et al.[14], who created a multi-modal transport model (which include the hinterland and maritime transport components) to simulate maritime freight flows (container, RoRo, bulk, liquid, general cargo) between 333 centroids globally. The OxMarTrans model is inspired by these previously developed models but includes several refinements and extensions; (1) we simulate flows between subnational units (3,380 centroids) instead of country centroids, (2) we include a simplified multi-model hinterland representation in the model, (3) we embed an observed maritime transport network, that consist of route connections between around 1400 ports globally, in the model to take revealed route choice decisions into consideration, (4) we add estimated sector-specific capacities to all ports and transportation routes, such that capacity constraints can be included in the model, and (5) we perform a sector-specific flow allocation (11 sectors) such that allocated flows can be linked to a multi-regional input-output table.

The model descriptions covers five components: (1) the origin-destination flow allocation, (2) the hinterland transport network representation, (3) the maritime transport network representation, (4) cost function, and (5) the flow allocation procedure. Moreover, we perform a validation of the model output.

Origin-destination flow allocation

Global country-to-country maritime trade flows, both in value and weight terms, per sector are derived from the global modal split model (see Supplementary Note 1: Modal Split Model). These flows will be routed on the hinterland and maritime transport networks between a number of origin (O) and destination (D) locations. Here, centroids of subnational administrative boundaries are taken as OD locations. We use administrative boundaries from the Database of Global Administrative Areas (GADM)[6], for which we extract the second administrative boundary layer per country, except for the United Kingdom for which we extract the third administrative boundaries. For countries without lower administrative boundaries (e.g. some small islands), we use the first administrative boundary layer. We use population data (CIESIN Gridded Population of the World, version 4[7] at 30 arcseconds) to assign a population weight to the centroids. We use the weight to determine the size of the trade flow between two regions. Similar as in Martinez et al.[14], Maritime trade flows (F) from administrative region (i) in the origin *(O)* and destination country *(D*) for a given sector *(s)* are found by scaling the total trade flow between the two countries with the admin population (P):

---

[6] https://gadm.org
[7] https://sedac.ciesin.columbia.edu/data/set/gpw-v4-population-density-rev11

$$F_{O_i,D_j,s} = F_{O,D,s} * \frac{P_{O_i}}{P_O} * \frac{P_{D_j}}{P_D}$$

Per maritime trade flow between two centroids, the OD database contains information on the quantity (in tonnes) and value (in USD) shipped.

Hinterland transport network representation

The hinterland transport network consist of a multi-modal (road, rail, and inland waterways transport (IWW)) network representation that connects centroids to relevant ports. We do not aim to accurately simulate hinterland transport routes choices, but primarily aim to represent the integration of ports in the hinterland transport network and the availability of different modes to ship goods from port to centroid, as both aspects guide port choice decisions.

Centroids are connected to ports via the road, rail and IWW networks (if this option is feasible). We only connect centroids to domestic ports and ports in neighbouring countries, except for landlocked countries where we also connect centroids to ports in the neighbours of neighbouring countries. Centroids of island states are only connected to domestic ports, where we assume that a short-sea shipping network exist to import and export goods from smaller islands (in case an island nation consist of a number of smaller islands) to the main ports of the island states.

We use the global road and rail network, similar as described in Supplementary Note 1 to make the connections between admin regions and ports. For the IWW network, we extract global river network data from HydroRivers data[8] and the European IWW network from UNECE[9], and combine this with a global river port database from WorldPortSource[10]. Centroids are connected to river ports and railway nodes via the road network. We end up with a hinterland transport network that connects ~160,000 unique centroid-port pairs with one another.

Maritime transport network representation

The maritime network consist of a network of ports and feasible maritime transport routes between port pairs. We have mapped the geographical location (lat/lon) of 1400 ports and derived information on the estimated turnaround time (time spend in port), the number of port calls, and port capacities per sector from Automatic Identification System (AIS) data in previous work[19]. Moreover, by analysing the sequence of port calls of around 10,000 vessels over time (between 2019 – 2020), we have derived a maritime transport database of connections between ports and the associated route capacity (based on the utilized capacities of the vessel). In total, the maritime transport database contains information on the distance and vessel capacity between ~150,000 port pairs in the global maritime transport network. To allocate this information on a routable network, we use the Oak Ridge National Laboratory shipping network, similar as in Supplementary Note 1, and link ports to the routable maritime network. The use of observed maritime transport information allows us to accurately simulate the port

[8] https://www.hydrosheds.org/page/hydrorivers
[9] https://unece.org/where-navigate-network-inland-waterways-europe-and-its-parameters
[10] http://www.worldportsource.com/index.php

choice for trade between two country countries based on the integration (i.e. connectivity, capacity) of various ports in the maritime transport network. Moreover, it allows predicting transhipment flows between country pairs.

Different vessel types are being used to ship goods for the different sectors, which all have different costs, travel times, port capacities, unloading times, dwell time, and handling cost. Moreover, given that they use different terminals within ports, the transport networks do not necessarily interact with one another. Therefore, within the maritime network, we distinguish between five broad vessel types; Container, General Cargo, Liquid bulk, RoRo or Dry Bulk vessels. Based on an conversion table of vessel types to economic sectors derived in earlier work[19], we can estimate what fraction of the capacity per vessel type is being used to ship goods for a certain sector. Therefore, the maritime transport database is also split between these five vessel types and the total maritime transport database contains information on ~280,000 port pair connections across the five vessel types.

At every port, a capacity is added per vessel type based on available AIS data. Port capacity is non-trivial, as many definitions exist (e.g. maximum instantaneous capacity, maximum annual capacity, optimum annual capacity), and there is no agreed standard definition, nor a global database, on port capacity. Here, we derive a globally consistent estimate of what we call the maximum operational capacity (MOC) of the port; the operational capacity which a port can theoretically reach within existing operational capabilities. First, per vessel type, we estimate the utilization rate (UR) of the port by looking at the weekly port capacity called at ports (multiply the number of calls with the deadweight tonnage of the vessels), and dividing the median with the maximum weekly port capacity called. We then estimate the yearly trade flow (TF) (incoming and outgoing flows) per port and vessel type using the methodology discussed in previous work using AIS data over the period 2019-2020[19]. The TF is then divided by the UR rate to derive the MOC per port (p), vessel type (t) and flow direction (f):

$$MOC_{p,t,f} = \frac{TF_{p,t,f}}{UR_{p,t}}$$

Cost function

We add a freight cost to every network segment in our combined hinterland and maritime transport network. The cost function used is similar as the one used in Supplementary Note 1, except that we add an additional value of time in the cost function instead of estimating this separately as done in the Modal Split Model. The total freight cost (C) per mode (m) and sector (s):

$$C_{m,s} = D_{c,m}d_m + T_{c,m}t_m + H_{c,m} + \ VC\ VOT_s(t_m + L_m)$$

with *Dc* the distance costs (in dollar per tonnes per km), *d* the distance (km), *Vc* the value to tonnes conversion (USD per tonnes), *Tc* the time costs (in dollar per tonnes per km), *t* the transit

time (in hours), *VOT* the value of time (in dollar per tonnes per hour), *L* the (un)loading and dwell time (in hours) and *Hc* the handling costs (in dollar per tonnes). The cost elements included capture the different components that together determine freight costs. However, we still ignore important factors that could not be included because of data limitations, such as costs associated with imbalances/empty legs or other route varying freight cost components, which lead to high freight costs in some regions (e.g. Pacific islands).

VC is taken from the output of the Modal Split model. The VOT metric is taken as a percentage of the value of the good to be transported in line with the findings of Hummels and Schaur[10]. We set a VOT value per economic sector based on Hummels and Schaur[10] and De Jong et al.[6], which are summarised in Supplementary Table 8.

For the hinterland transport network, speed, time and distance costs are similar as used in Supplementary Note 1 and summarised in Supplementary Table 4-7. Dwell times and handling are used when transferring goods from one mode to the other, with values used summarised in Supplementary Table 4. Moreover, if ports in other countries are used, the border crossing times are added as additional dwell time (see Supplementary Note 1). The time costs are found by dividing the distance with the mode-specific speed (Supplementary Table 3) and applying the time costs to this.

For the maritime transport network, we add speed, time and distance cost per vessel type, as shown in Supplementary Table 3-7. Port handling costs vary per good and across ports. We add vessel specific port handling costs to the individual port based on a database provided by the ITF-OECD (not publicly available), which is constructed as part of the ITF global freight model development[14]. The total dwell time of goods at the port consist of the unloading/loading time and the dwell of cargo in the port (time between entering/leaving on the land-side and loading/unloading). The loading/unloading time is derived from AIS data, for which we take the median turnaround time of the different vessel types in a port. The dwell time for containers is set to 3 days for Group 1 countries and 6 days for Group 2 countries[14], but can be substantially higher for countries in Sub-Saharan Africa (up to 16 days). The dwell times of goods transported by other vessel types is more context-specific, but we use number as found in the literature. In general, we set dwell time in Group 2 countries as twice that of Group 1 countries. For transhipment, we assumes that the handling costs are 75% of the handling costs of importing/exporting. Transhipment dwell are commonly longer than importing/exporting[20,21], and we apply a factor 1.2 to the dwell times for importing/exporting.

Flow allocation procedure

The flow allocation is done by means of a capacity-constraint (all-or-nothing) shortest path routing approach using Dijkstra's shortest path algorithm[15]. We follow a step-wise approach too allocation flows per economic sector:

1) Extract the sector-specific maritime trade flows between country pairs and disaggregate the flows to the centroids.
2) Calculate the fraction of the five main vessel types for the economic sector and add the capacities at ports and maritime routes, the handling cost, dwell time, and time and distance costs to the maritime transport network. Moreover, select the VOT for the economic sector.
3) Loop over the country pairs and allocate the trade flows between country centroids using the shortest path algorithm. If flows are allocated, reduce the capacity in the port nodes and on the maritime transport legs until capacity is reached.
4) Repeat until all flows are allocated and store the all paths between O and D.
5) In case certain ports are underutilised (allocation lower than port capacity) and others are overutilized (allocated more than port capacity), rebalance the allocation such that the error between the initial allocation and the port capacities is minimized.
6) Repeat for the every economic sector, resulting in a flow allocation per economic sector.

Model validation

Validating the output of the transport model is complex, as there is no external dataset available to validate port-to-port trade flows. We therefore perform two types of external validation; (1) compare the distribution of trade across ports for four countries we have official data for (United States, United Kingdom, Japan, New Zealand), and (2) evaluate the flows through the Suez and Panama canal.

The aim of the model is to evaluate the share of maritime that is going in and out the ports to meet a countries import and export. We collected data for four countries (United States, United Kingdom, Japan, New Zealand), and derive, per sector, the cumulative distribution of maritime trade across the country's ports and compared this to the model output. Results are shown in Supplementary Fig. 11, showing an excellent fit for the United Kingdom and the United States. For New Zealand, all sectors show a good agreement, except the export of 'Other Manufacturing' products. For Japan, the all sectors show a good agreement, except the imports of 'Textiles and wearing apparel' and the exports of 'Food and Beverages'. However, given all model uncertainties, the comparison shows that the model is able to distribute sector-specific imports and exports to the correct ports.

In addition, we evaluate whether the flows through the Panama and Suez canal are in line with reality, which gives us an indication whether the flow distribution between ports globally is realistic. Our model estimates that around 1.1 trillion USD (13.8% of maritime trade in value terms) and 1.04 billion tonnes (10.8% of maritime trade in quantity terms) flows through the Suez canal. According to official records, more than 1 trillion USD goes through the Suez canal

every year, which equalled 998 million tonnes in 2015[11]. Moreover, we predict that around 490 million USD (6.2% of maritime trade in value terms) and 461 million tonnes (4.8% of maritime trade in quantity terms) flows through the Panama canal every year. According to the Panama Canal Authority, in 2015 reported a flow of 340 million tonnes through the canal and 470 million tonnes in 2019[12]. The top 10 countries using the Panama canal in 2021 are reported to be the United States, China, Japan, South-Korea, Chile, Mexico, Peru, Colombia, Canada and Ecuador[13]. These 10 countries are also the top 10 countries our model simulation predicts in terms of quantity (through slightly different order).

[11] https://www.suezcanal.gov.eg/English/Navigation/Pages/NavigationStatistics.aspx
[12] https://www.pancanal.com/eng/general/reporte-anual/index.html
[13] https://www.pancanal.com/eng/op/transit-stats/2021/Table-10.pdf

Supplementary Figures

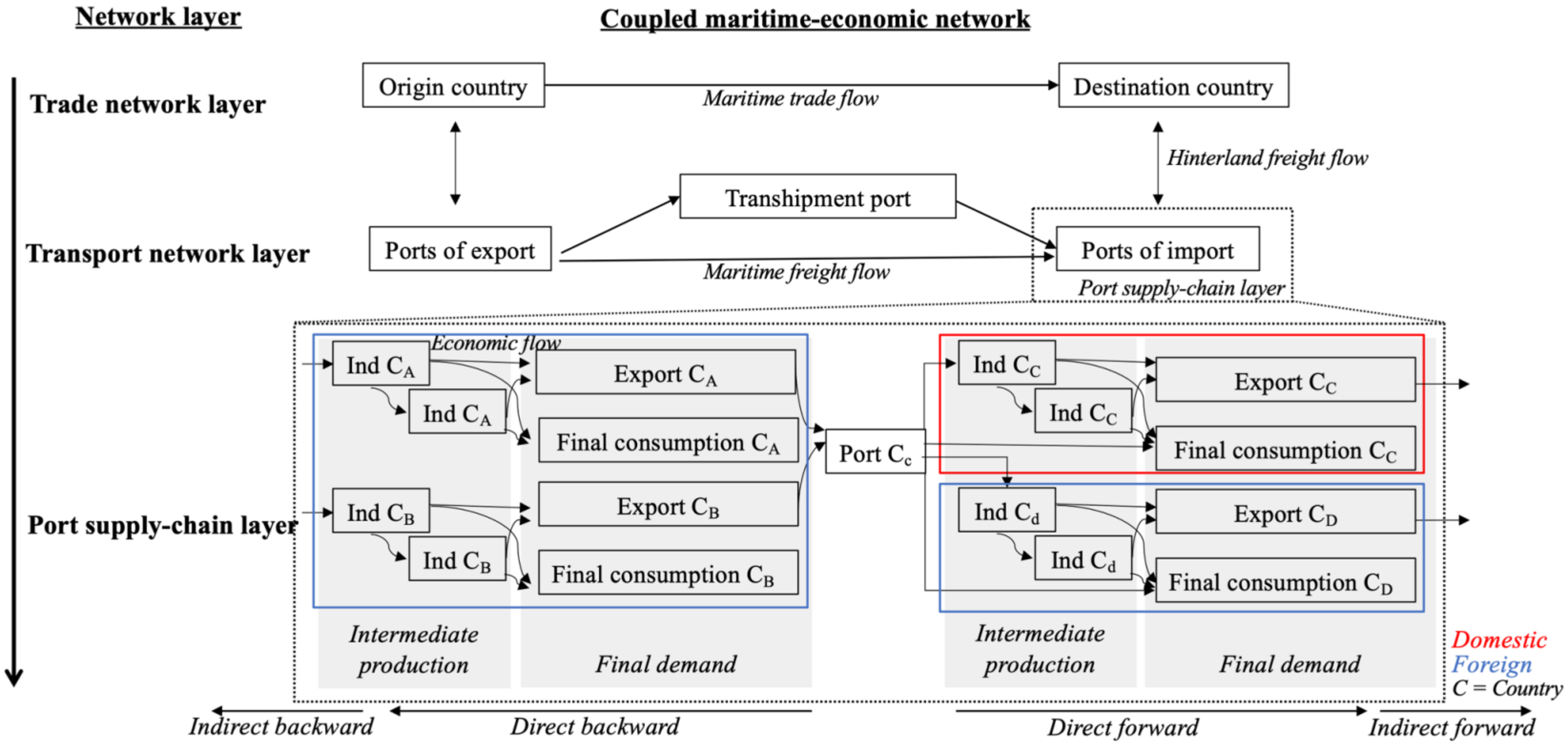

Supplementary Fig. 1 Conceptual framework multi-layered maritime-economic network. The top layer shows the 'Trade network layer', which is constructed using the modal split model. The layer describes the size of the maritime trade flow between two countries. The middle layer shows the 'Transport network layer', which describes the freight flow allocation on the maritime and hinterland transport network. The bottom layer is the 'Port supply-chain layer' which describes how economic flows going in and out ports are used in the economy, which is done on a port by port basis. This layer shows an example port in country C (Port Cc) that receives goods from country A and B, which were produced using intermediate production (Ind) in these countries. The flows going through port Cc are then being used as intermediate products in country C (which are then further exported or consumed) or go directly into final consumption in country C. Alternatively, it could be that the flows are going to another country, country D, which is dependent on port C via an hinterland transport network. We further distinguish between domestic economic flows (e.g. flows between the port and the country it is located it) or foreign economic flows (flows coming from or going to foreign economies). Similarly, we can distinguish between direct and indirect backward/forward flows. Direct flows feed/come out of the port directly, while economic flows further downstream/upstream in the supply chain are considered indirect flows.

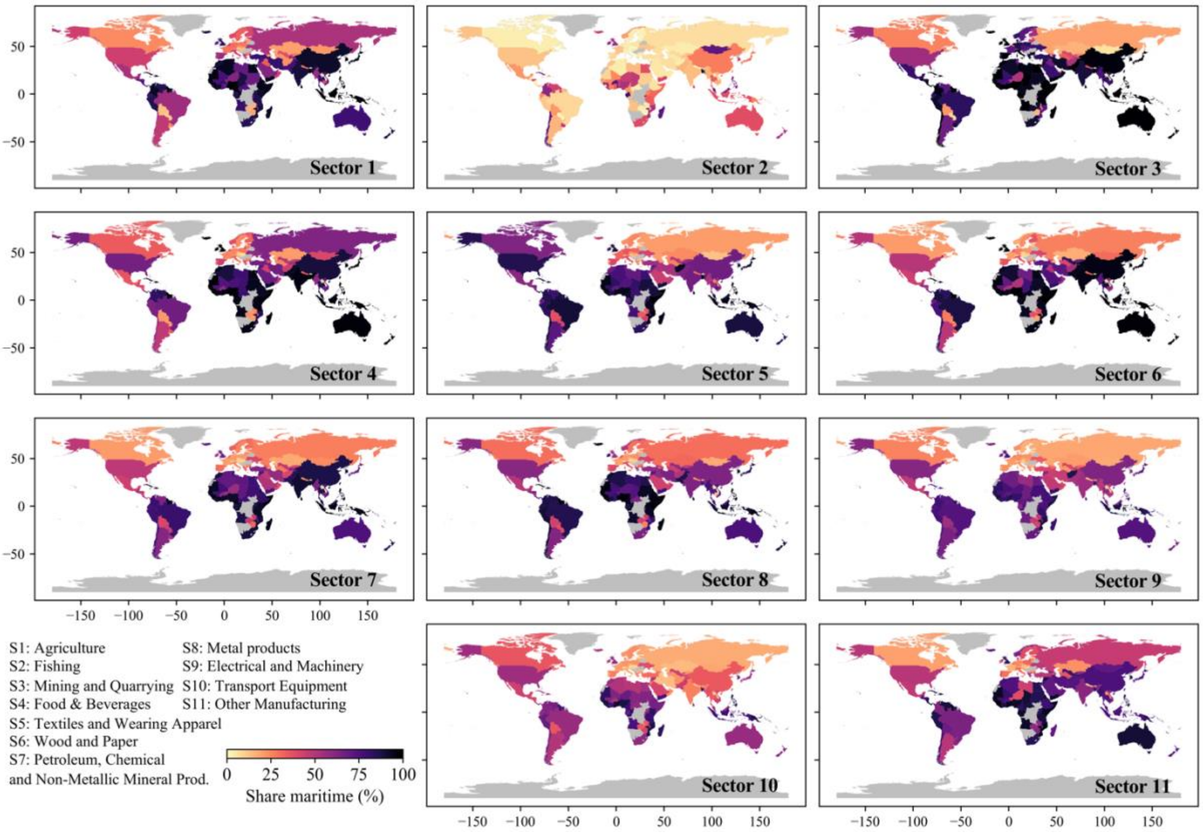

Supplementary Fig. 2 Share of maritime trade in country imports. The share of maritime transport in total imports for the 2015 trade network. Each panel shows the percentages for a particular economic sector. Grey indicates no data.

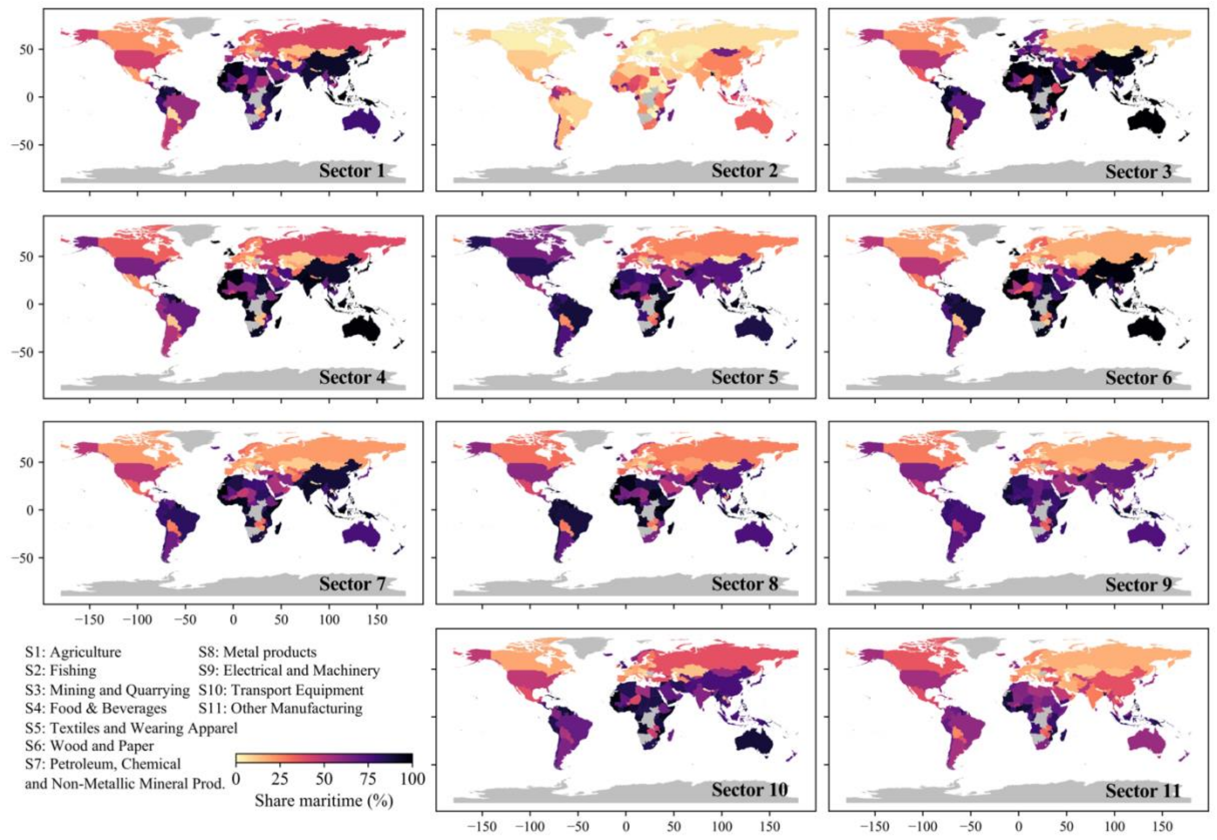

Supplementary Fig. 3 Share of maritime trade in country exports. The share of maritime transport in total exports for the 2015 trade network. Each panel shows the percentages for a particular economic sector. Grey indicates no data.

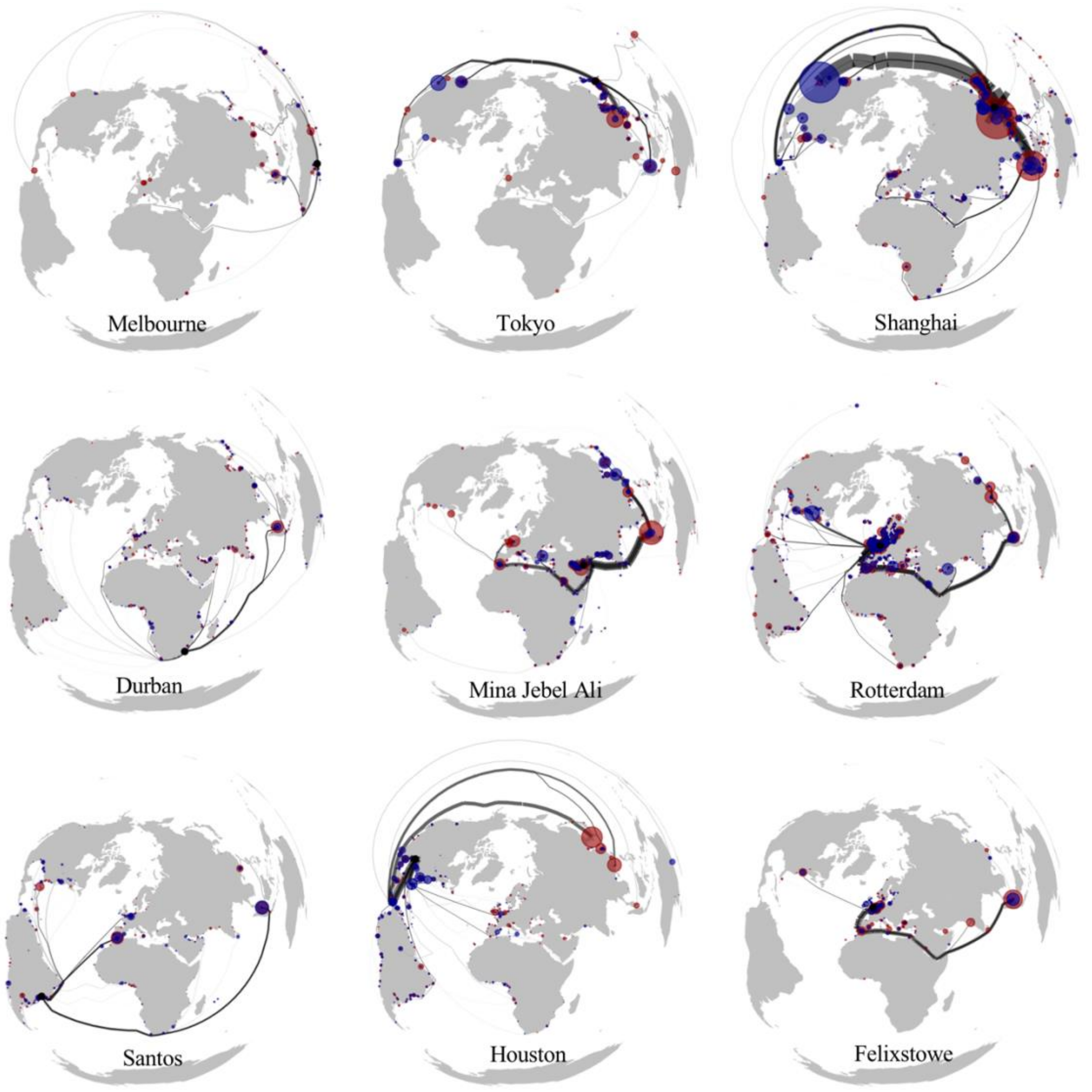

Supplementary Fig. 4 Examples of port's spatial connectivity. The top 200 largest importing (blue, left) and exporting (red, right) trade flows to and from a port, including the location of the origin/destination port. Examples are shown for 9 ports globally.

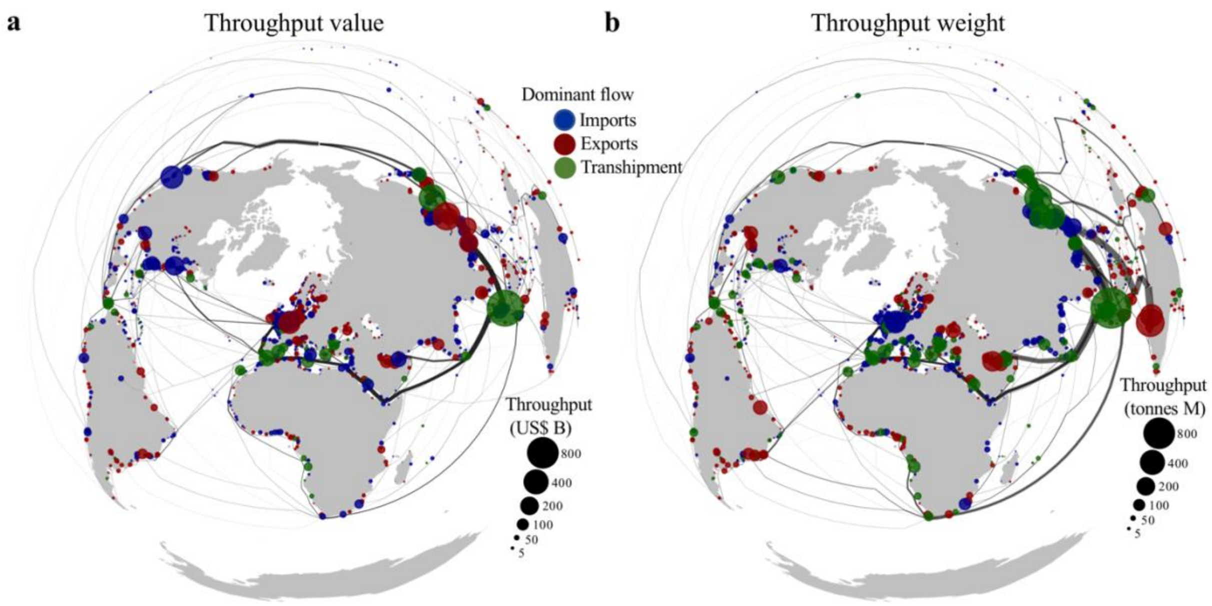

Supplementary Fig. 5 Global maritime transport network. (a) The global maritime transport network, consisting of the port-level throughput and dominant flow direction, and the maritime transport routes used. Size of dots and line thickness related to value flows through a port or on a route. (b) Same as (a) but expressed in quantity terms.

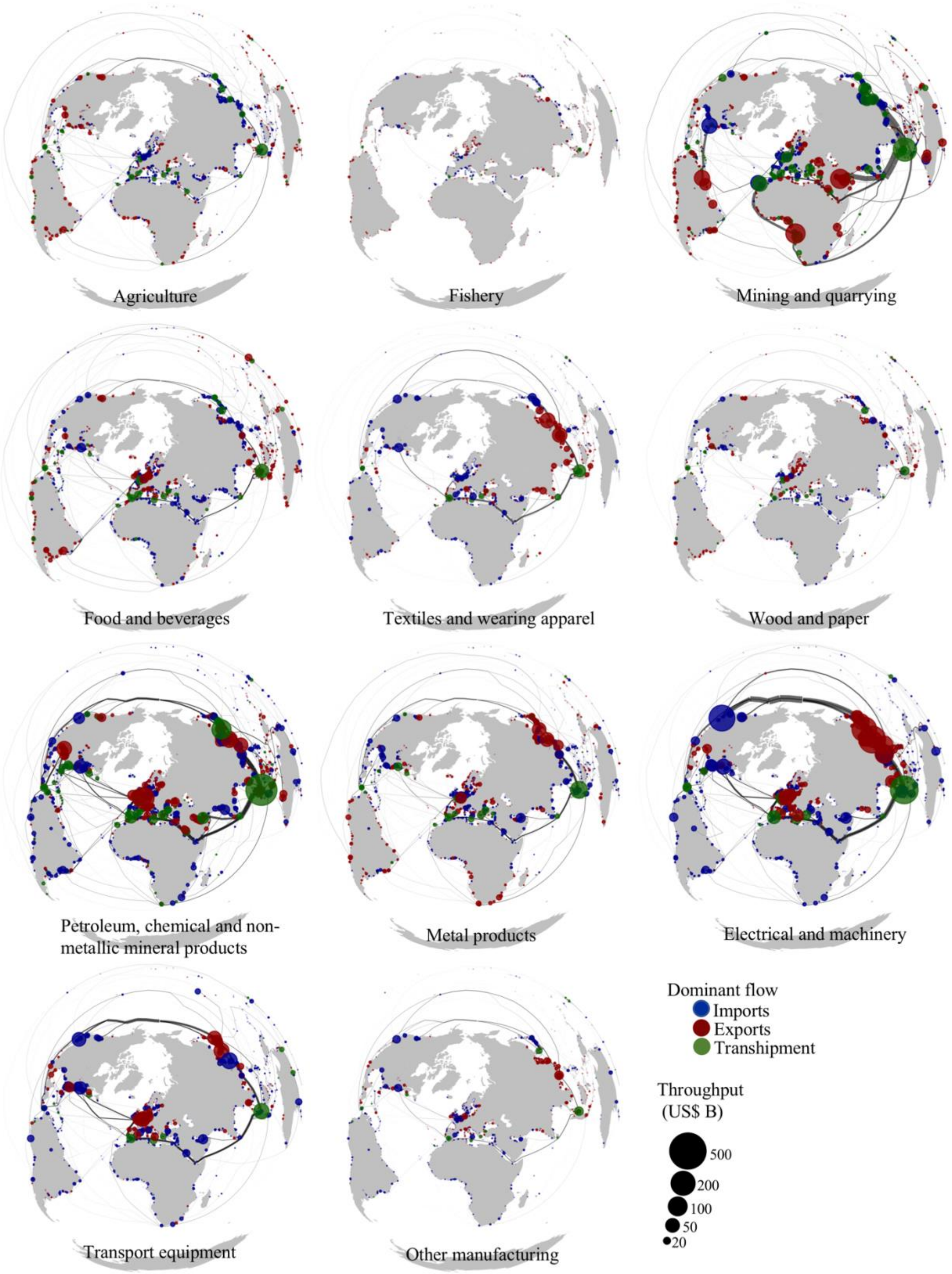

Supplementary Fig. 6 Global maritime transport network per industry sector. The global maritime transport network, consisting of the port-level throughput and dominant flow direction, and the maritime transport routes used per economic sector. Size of dots and line thickness related to value flows through a port or on a route.

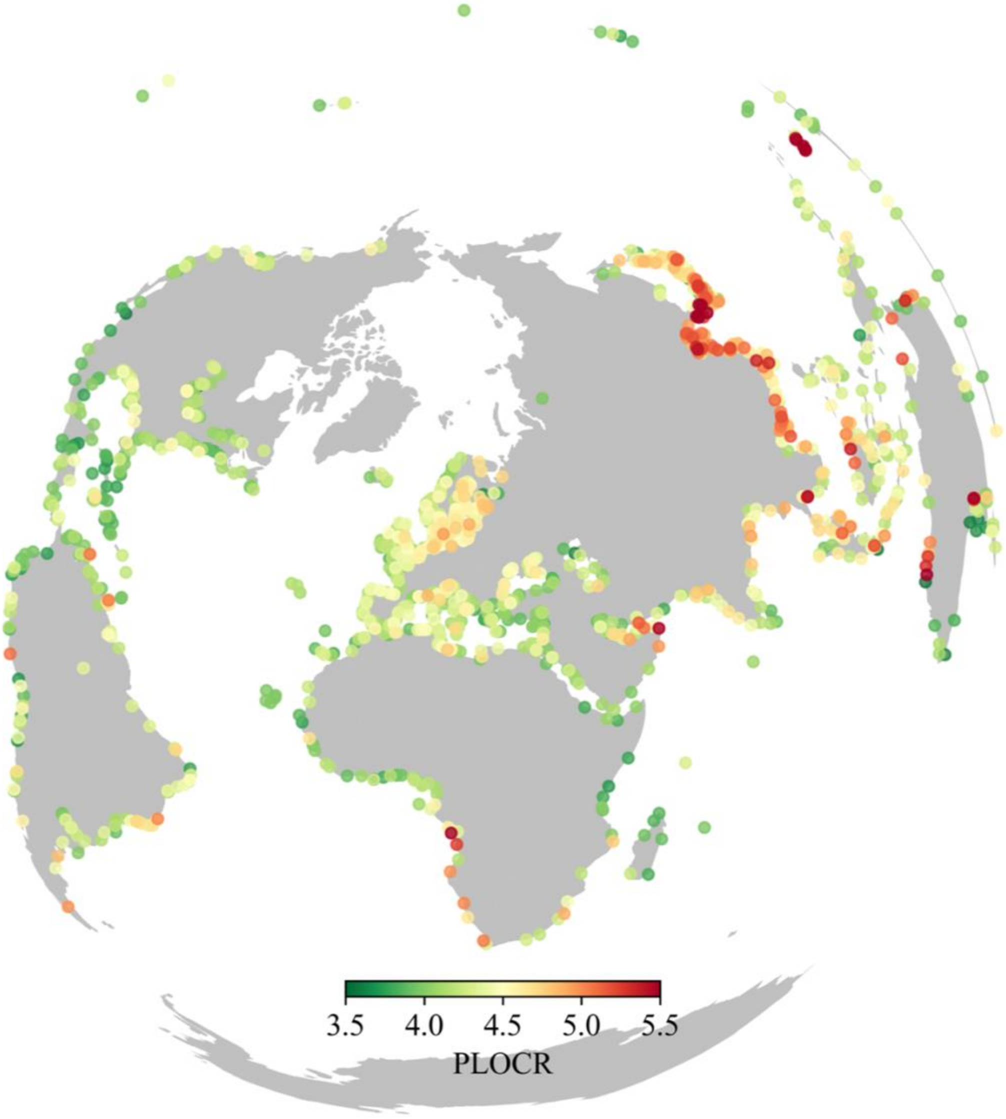

Supplementary Fig. 7 Geographical distribution of PLOCR values. The geographical location of ports with their associated relative port-level output coefficient (PLOCR), indicating the amount of industry output embedded in every dollar trade flow going through a port.

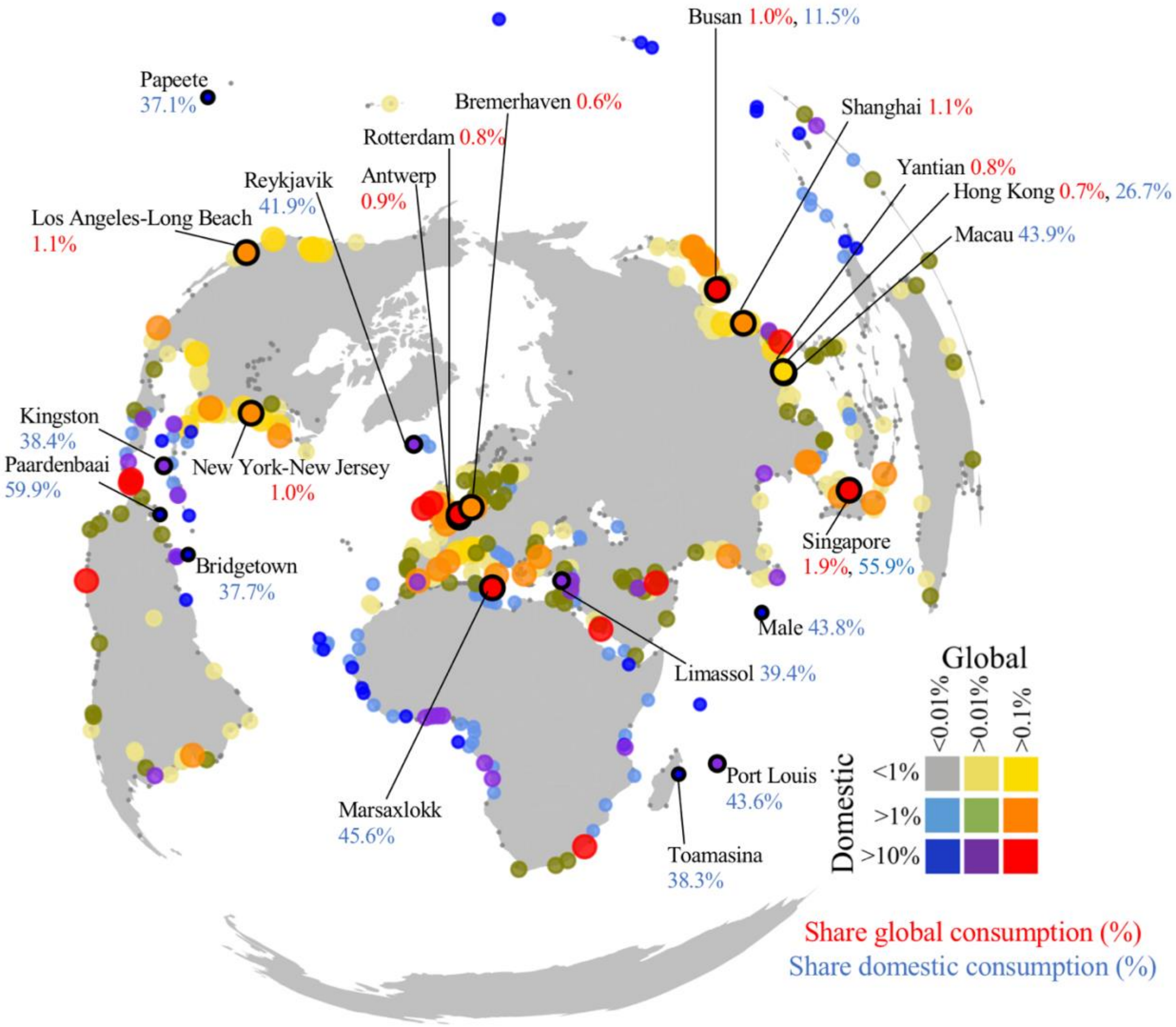

Supplementary Fig. 8 Distribution of the domestically and globally critical ports in terms of final consumption. The importance of trade flows going through ports in terms of its contribution to the domestic consumption as a percentage of total domestic consumption and global consumption as a percentage of total global consumption. The ten ports most critical ports in terms of domestic and global consumption are highlighted together with the associated percentage value (domestic in blue, global in red).

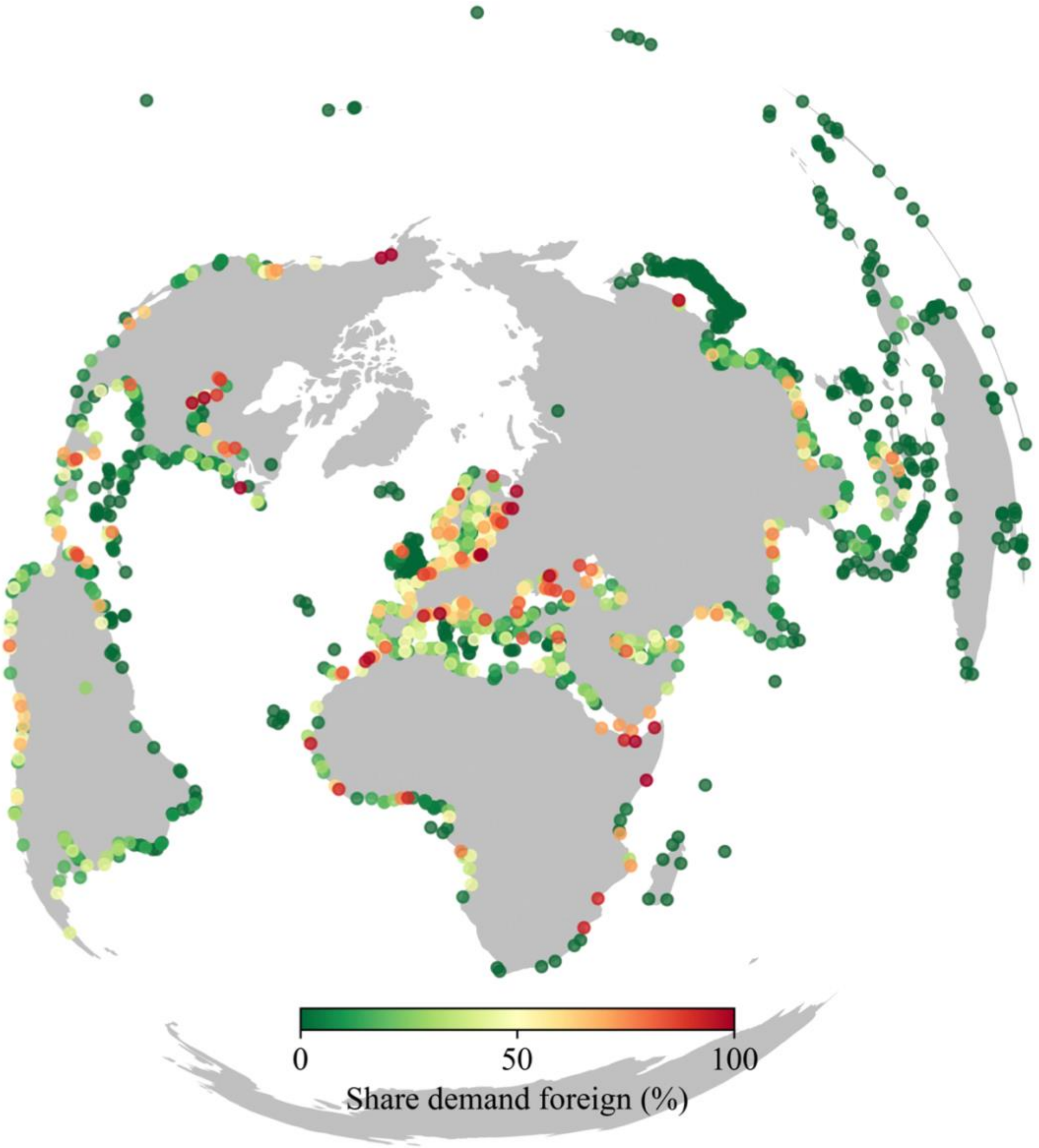

Supplementary Fig. 9 Share of foreign demand in port-level import coefficient. The share of port-level import coefficient that is linked to a foreign economy because of land-based connections.

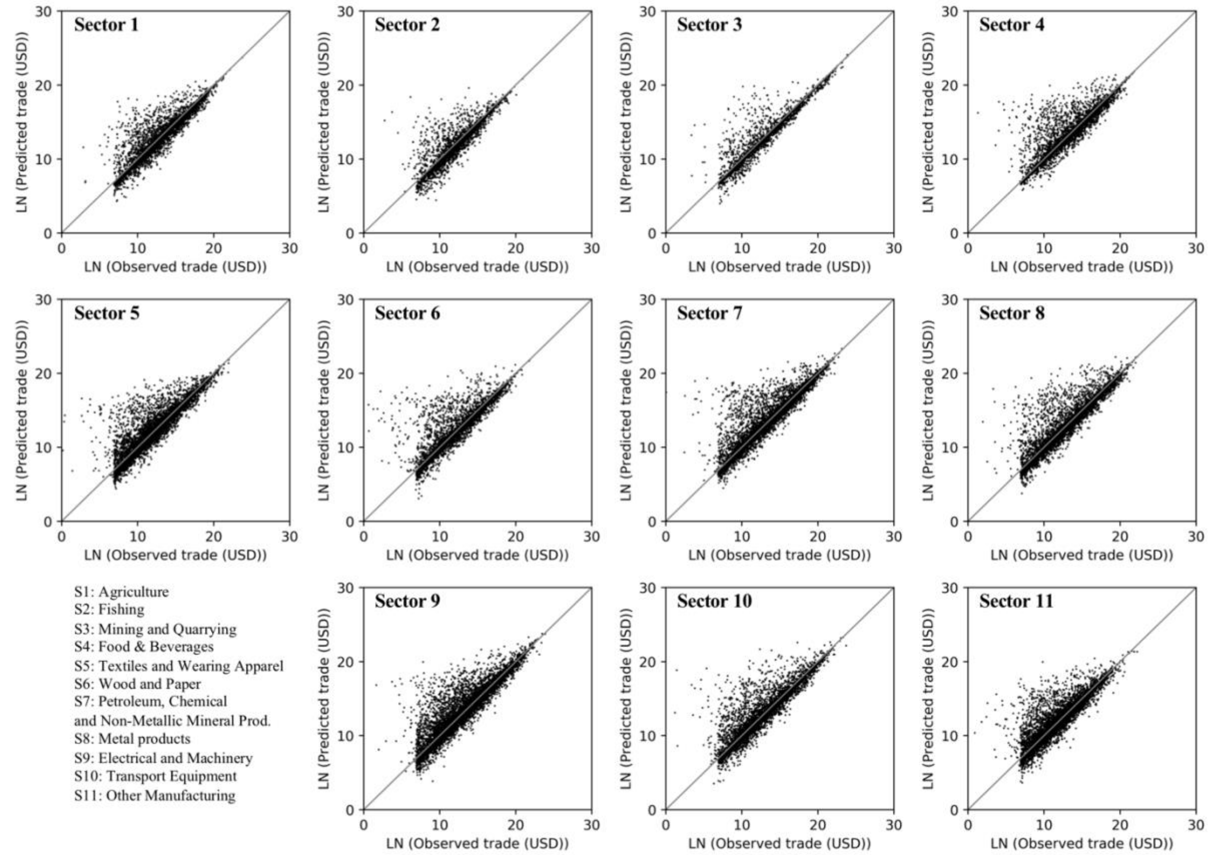

Supplementary Fig. 10 Validation modal split model. Validation plots showing the actual maritime trade flows based on UN Comtrade data[2] and predicted maritime trade flows using the mode prediction model.

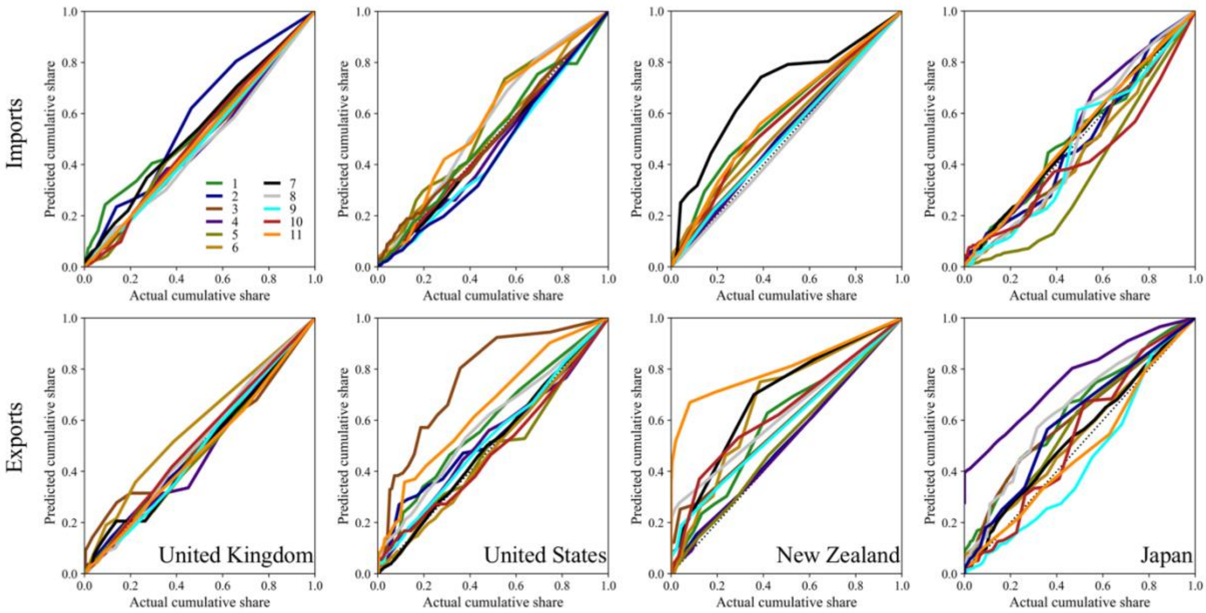

Supplementary Fig. 11 Validation trade distribution. Validation plots showing the actual distribution of trade over the ports in a country and the predicted distribution based on the maritime transport model. This is based on detailed port-level, sector-specific, trade flows which are available for four countries; United Kingdom, United States, New Zealand and Japan, United States. The top panel represent imports, whereas the bottom panel represent exports.

Supplementary Tables

Supplementary Table 1. Overview of economic sectors used in this study.

Sector	Description
1	Agriculture
2	Fishing
3	Mining and Quarrying
4	Food & Beverages
5	Textiles and Wearing Apparel
6	Wood and Paper
7	Petroleum, Chemical and Non-Metallic Mineral Products
8	Metal Products
9	Electrical and Machinery
10	Transport Equipment
11	Other Manufacturing

Supplementary Table 2. Overview of the concentration of port-level trade per sector. The number represent the number of ports (out of the 1380 ports) that contribute to 10%, 50% and 90% of trade (imports and exports).

	**Import**			**Export**		
**Sector**	10%	50%	90%	10%	50%	90%
1	5	60	344	3	42	267
2	2	24	168	2	33	217
3	2	33	199	1	19	188
4	5	74	392	5	50	309
5	2	19	128	1	7	92
6	4	39	221	3	29	187
7	4	72	371	3	46	294
8	4	41	272	3	38	236
9	1	22	177	2	17	124
10	3	27	181	2	15	124
11	2	19	142	1	13	99
All	4	56	378	3	48	366

Supplementary Table 3. Speed per transport mode used in modal split model and transport model

Mode	Group	Parameter	Group 1 countries	Group 2 countries	Source
Air		Speed (km/h)	750	750	[14]
Road	Primary road	Speed (km/h)	80	70	[14]
	Secondary road	Speed (km/h)	65	55	[14]
	Beltway/national road	Speed (km/h)	40	30	[14]
	Tracks/unpaved roads	Speed (km/h)	10	5	[14]
Maritime	Container	Speed (km/h)	30	30	[21]
	Dry Bulk	Speed (km/h)	20	20	[21]
	General Cargo	Speed (km/h)	22	22	[21]
	RoRo	Speed (km/h)	28	28	[21]
	Liquid bulk	Speed (km/h)	20	20	[21]
IWW		Speed (km/h)	8	8	[14]

Note: Group 1 countries include European countries, Japan, Australia, Canada and United States. Group 2 countries include the rest of the world.

Supplementary Table 4. Dwell time and handling costs hinterland network

From	To	Dwell time (h)	Handling cost (USD per tonnes)	Source
Centroid	Road	10	4	[6], [14]
Road	Rail	32	5	[6], [14]
River port	IWW	120	1	[6], [22]
Road	Port	50	4	[6], [14]
Rail	Port	50	5	[6], [14]
Port	IWW	120	1	[6], [22]
Road	Air	40	4	[6], [14]
Border	Border	Country-specific	0	[13]

Supplementary Table 5. Dwell times and handling costs at ports and airports

Mode	Group	Parameter	Group 1 countries	Group 2 countries	Source
Air		Handling cost (USD per tonnes)	450	450	[6]
		Dwell time (h)	4	48	[14]
		Transhipment dwell time (h)	18	18	[17]
Maritime	Container	Handling cost (USD per tonnes)	Port-specific	Port-specific	ITF-OECD
		Cargo dwell time (h)	3	6	[21]
		Vessel turnaround time (h)	Port-specific	Port-specific	AIS data
	Dry Bulk	Handling cost (USD per tonnes)	Port-specific	Port-specific	ITF-OECD
		Cargo dwell time (h)	15	30	[21]
		Vessel turnaround time (h)	Port-specific	Port-specific	AIS data
	General Cargo	Handling cost (USD per tonnes)	Port-specific	Port-specific	ITF-OECD
		Cargo dwell time (h)	8	16	[21]
		Vessel turnaround time (h)	Port-specific	Port-specific	AIS data
	RoRo	Handling cost (USD per tonnes)	Port-specific	Port-specific	ITF-OECD
		Cargo dwell time (h)	5	10	[21]
		Vessel turnaround time (h)	Port-specific	Port-specific	AIS data
	Liquid bulk	Handling cost (USD per tonnes)	Port-specific	Port-specific	ITF-OECD
		Cargo dwell time (h)	2	4	[21]
		Vessel turnaround time (h)	Port-specific	Port-specific	AIS data

Note: Group 1 countries include European countries, Japan, Australia, Canada and United States. Group 2 countries include the rest of the world.

Supplementary Table 7. Distance and time costs per transport mode

Mode	Group	Parameter	Value	Source
Air		Distance costs (USD per t-km)	0.2	[23]
		Time costs (USD per t-h)	135	[23]
Maritime	Container	Distance costs (USD per t-km)	0.002	[23]
		Time costs (USD per t-h)	0.03	[23]
	Dry Bulk	Distance costs (USD per t-km)	0.004	[23]
		Time costs (USD per t-h)	0.06	[23]
	General Cargo	Distance costs (USD per t-km)	0.002	[23]
		Time costs (USD per t-h)	0.03	[23]
	RoRo	Distance costs (USD per t-km)	0.002	[23]
		Time costs (USD per t-h)	0.03	[23]
	Liquid bulk	Distance costs (USD per t-km)	0.006	[23]
		Time costs (USD per t-h)	0.11	[23]
Road		Distance costs (USD per t-km)	Country-specific	ITF-OECD
		Time costs (USD per t-h)	4	[23]
Rail		Distance costs (USD per t-km)	Country-specific	ITF-OECD
		Time costs (USD per t-h)	1	[23]
IWW		Distance costs (USD per t-km)	Country-specific	ITF-OECD
		Time costs (USD per t-h)	0.2	[23]

Supplementary Table 8. Value of time (VOT) expressed as the percentage of product value per day

Sector number	Description	VOT (%)
1	Agriculture	1.0
2	Fishing	3.1
3	Mining and Quarrying	0.5
4	Food & Beverages	3.1
5	Textiles and Wearing Apparel	1.0
6	Wood and Paper	1.0
7	Petroleum, Chemical and Non-Metallic Mineral Products	1.0
8	Metal Products	1.0
9	Electrical and Machinery	2.0
10	Transport Equipment	4.3
11	Other Manufacturing	2.0

Supplementary References

1. de Jong, G. Mode Choice Models. *Model. Freight Transp.* 117–141 (2013). doi:10.1016/B978-0-12-410400-6.00006-9
2. United Nations Statistical Division. UN Comtrade database. *UN Comtrade database* (2020). Available at: https://comtrade.un.org. (Accessed: 1st February 2020)
3. Gaulier, G. & Zignago, S. BACI : International Trade Database at the Product-level The 1994-2007 Version. (2010).
4. Ben-Akiva, M. & de Jong, G. The Aggregate–Disaggregate–Aggregate (ADA) Freight Model System. *Recent Dev. Transp. Model.* 117–134 (2008). doi:10.1108/9781786359537-007
5. Ben-Akiva, M. & Bierlaire, M. Discrete Choice Methods and their Applications to Short Term Travel Decisions. in *Handbook of Transportation Science* 5–33 (1999). doi:10.1007/978-1-4615-5203-1_2
6. de Jong, G. *et al. Schatting BASGOED rapportage DP1*. (2010).
7. Ben-Akiva, M., Bolduc, D. & Park, J. Q. Discrete Choice Analysis of Shippers' Preferences. *Recent Dev. Transp. Model.* 135–155 (2008). doi:10.1108/9781786359537-008
8. World Bank. World Development Indicators. *World Development Indicators Online Database* (2020). Available at: https://data.worldbank.org/data-catalog/world-development-indicators. (Accessed: 1st April 2020)
9. Mayer, T. & Zignago, S. The GeoDist Database on Bilateral Geographical Information. *Cepii* 18 (2011). doi:10.2139/ssrn.1994531
10. Hummels, D. L. & Schaur, G. Time as a trade barrier. *Am. Econ. Rev.* **103**, 2935–2959 (2013).
11. Hall, O., Bustos, M. F. A., Olén, N. B. & Niedomysl, T. Population centroids of the world administrative units from nighttime lights 1992-2013. *Sci. Data* **6**, 1–8 (2019).
12. Center for International Earth Science Information Network - CIESIN - Columbia University & Information Technology Outreach Services - ITOS - University of Georgia. Global Roads Open Access Data Set, Version 1 (gROADSv1). (2013).
13. World Bank. *Doing Business 2020: Comparing Business Regulation in 190 Economies*. **6**, (Washington, DC: World Bank, 2020).
14. Martínez, L. M., Kauppila, J. & Castaing, M. International freight and related carbon dioxide emissions by 2050 new modeling tool. *Transp. Res. Rec.* **2477**, 58–67 (2015).
15. Dijkstra, E. W. A Note on Two Problems in Connexion with Graph. *Numer. Math.* **271**, 269–271 (1959).
16. Bombelli, A., Santos, B. F. & Tavasszy, L. Analysis of the air cargo transport network using a complex network theory perspective. *Transp. Res. Part E Logist. Transp. Rev.* **138**, 101959 (2020).
17. Meijs, L. J. J. A. GLOBAL AIR CARGO FLOWS ESTIMATION BASED ON O/D TRADE DATA. (Delft University of Technology, 2017).
18. Tavasszy, L., Minderhoud, M., Perrin, J. F. & Notteboom, T. A strategic network choice model for global container flows: Specification, estimation and application. *J. Transp. Geogr.* **19**, 1163–1172 (2011).
19. Verschuur, J., Koks, E. E. & Hall, J. W. Global economic impacts of COVID-19 lockdown measures stand out in high-frequency shipping data. *PLoS One* **16**, (2021).
20. Raballand, G., Refas, S., Beuran, M. & Isik, G. *Why Does Cargo Spend Weeks in Sub-Saharan African Ports? Why Does Cargo Spend Weeks in Sub-Saharan African Ports?* (The World Bank, 2012). doi:10.1596/978-0-8213-9499-1
21. Ligteringen, H. & Velsink, H. *Ports and Terminals*. (Delft University Press, 2012).
22. Blauwens, G. & Van de Voorde, E. THE VALUATION OF TIME SAVINGS IN COMMODITY TRANSPORT. *Int. J. Transp. Econ.* **15**, 77–87 (1988).
23. van der Meulen, S. *et al. Cost Figures for Freight Transport*. (2020).
